# Supplementary material for: A multichromatic colorimetric detection method for Vibrio parahaemolyticus based on Fe3O4-Zn-Mn nanoenzyme and dual substrates
Source: Microbiol Spectr. 2023 Dec 6;12(1):e03189-23. doi: 10.1128/spectrum.03189-23 (PMC10783063; doi:10.1128/spectrum.03189-23)
Supplement: Supplemental material — Fig. S1 to S7; Tables S1 and S2. [file spectrum.03189-23-s0001.docx]

**Supplementary Material**

**A multichromatic colorimetric detection method for *Vibrio parahaemolyticus* based on Fe_3_O_4_-Zn-Mn nanoenzyme and dual substrates**

Wenteng Qiao^1^, LuliangWang^1,2^, Kun Yang^1^, Yushen Liu^1,2,3^^*^, Quanwen Liu^1^, Feng Yin^4*^

^1^College of Food Engineering, Ludong University, Yantai 264025, Shandong, China

^2^Bio-Nanotechnology Research Institute, Ludong University, Yantai 264025, Shandong, China

3 Shandong Key Laboratory of Biochemical Analysis, College of Chemistry and Molecular Engineering, Qingdao University of Science and Technology, Qingdao 266042, China

4 Centers for Disease Control and Prevention, Laishan District, Yantai 264003, Shandong, China

*Corresponding Author, No. 186, Hongqi Middle Road, Zhifu District, Yantai City, Shandong Province. Yushen Liu [yushenlys@163.com](mailto:yushenlys@163.com); Block B, Budweiser International Building, 1295 Main Street East, Laishan District city, Yantai. 1282128466@qq.com

Wenteng Qiao and Luliang Wang contributed equally to this work. Author order was determined by division of work.


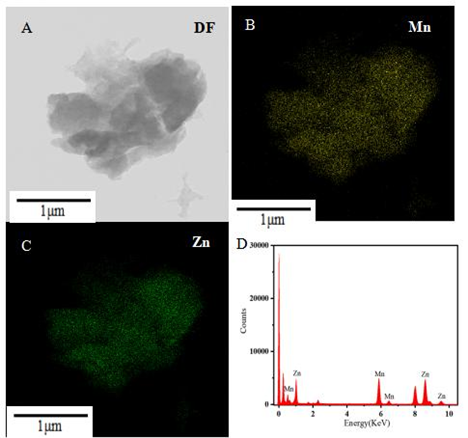


**FIG S1** Elemental mapping and analysis of Zn-MnO_2_ by TEM-energy-dispersive X-ray spectroscopy (EDS).


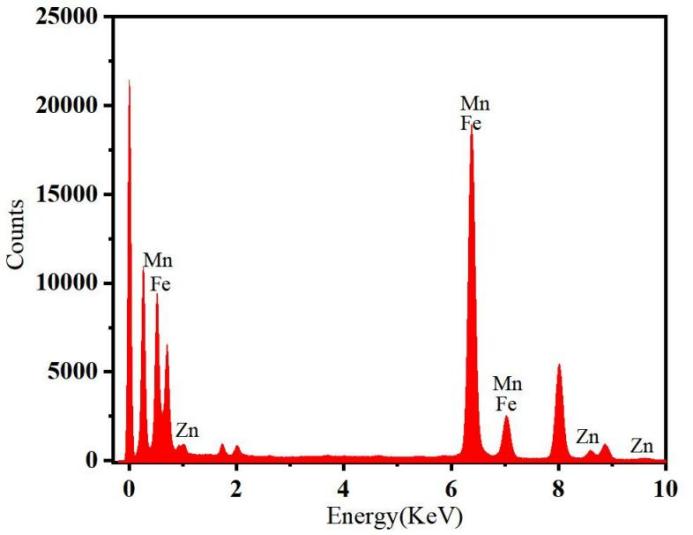


**FIG S2** Elemental energy spectrum of Fe_3_O_4_-Zn-Mn obtained by energy-dispersive X-ray spectroscopy (EDS).


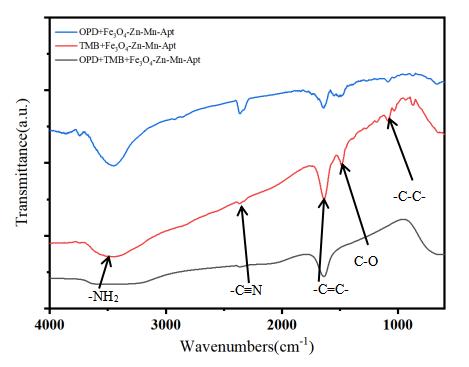


**FIG S3** Fourier-transform infrared (FTIR) analysis of the dual-substrate colorimetric system.

**INFORMATION OF THE BACTERIAL STRAINS**

**TABLE S1** Information of bacterial strains employed in this work

| **Bacteria** | **Abbreviation** | **ATCC No.** |
| --- | --- | --- |
| *Vibrio parahaemolyticus* | *V. parahaemolyticus* | 17802 |
| *Staphylococcus aureus* | *S. aureus* | 25923 |
| *Listeria monocytogenes* | *L. monocytogenes* | 19111 |
| *Salmonella typhimurium* | *S. typhimurium* | 14082 |
| *Escherichia coli O157:H7* | *E. coli O157:H7* | 25922 |

**BACTERIAL CUILTURE**

Supporting Information Table S1 lists the strains used, which are all stored in glycerol cryovials. They are recovered through routine bacterial culture. The bacterial concentration is determined using conventional plate counting and pour plate counting methods.


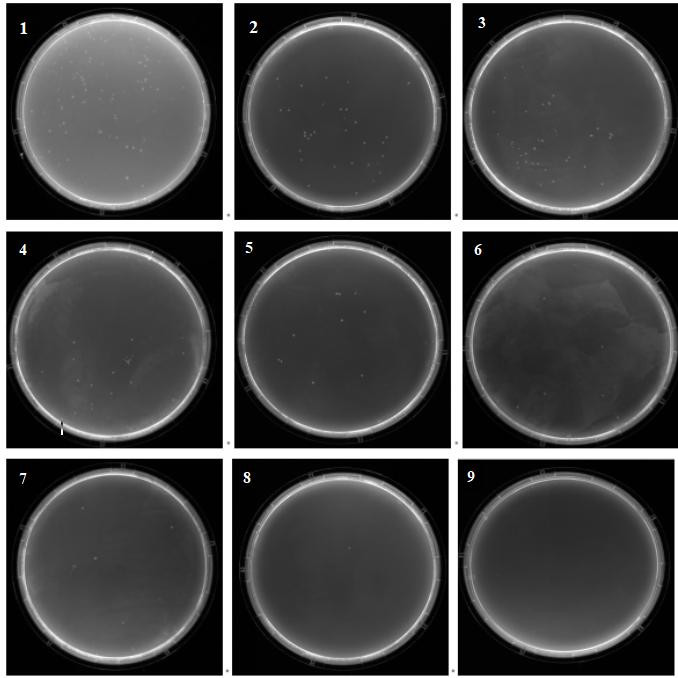


**FIG S4** The photo of the capture rate of *V. parahaemolyticus* by the Fe_3_O_4_-Zn-Mn-Apt probe.


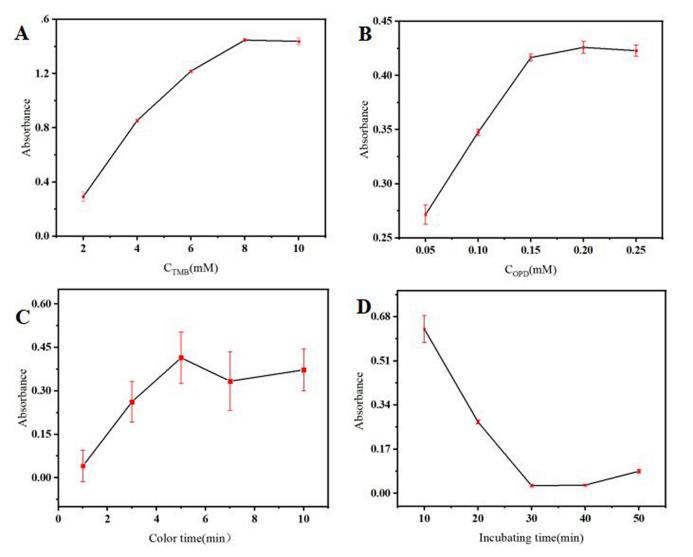


**FIG S5** Optimization experiment for conditions, TMB concentration (A), OPD concentration(B), Color time (C), Incubation time (D).


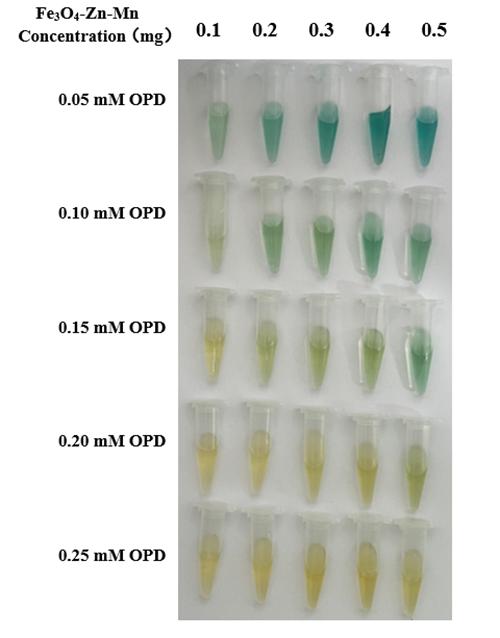


**FIG S6** Concentration optimization of dual substrate colorimetric detection system.

**
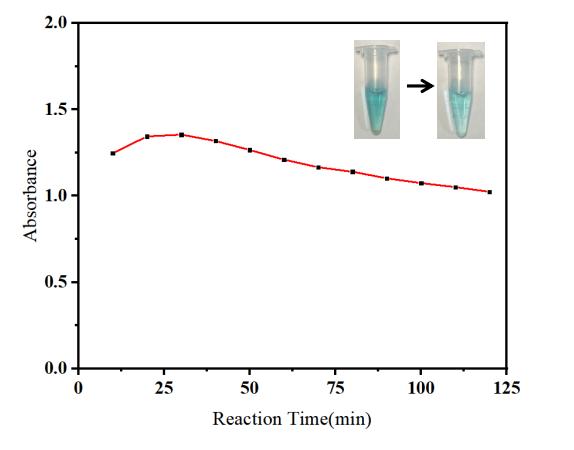
**

**FIG S7** Investigation of stability in colorimetric detection system.

**TABLE S2** Comparative Analysis of Methods for *V. parahaemolyticus* Detection.

| **Analytical**  **Methods** | **Samples** | **Linear range** | **Detection time** | **LOD** | **Ref.** |
| --- | --- | --- | --- | --- | --- |
| Flfluorescent reporting  Probes | clam | 10^2^-10^6^ cfu mL^-1^ | 150 min | 10^2^ cfu mL^-1^ | (1) |
| Nuclear Magnetic Resonance | shrimp | 10-10^6^ cfu mL^-1^ | — | 10^4^ cfu mL^-1^ | (2) |
| Loop-mediated isothermal amplifification | squids | f8–80 cfu mL^-1^ | 45min | 0.3 cfu 25g^-1^ | (3) |
| A monoclonal antibody | — | 1.3–1.6 × 10^6^ cfu 100μL^-1^ | 20min | 10^0^ cfu 100μL^-1^ | (4) |
| Dual-Functional Aptamers and Cut-Assisted Rolling Circle  Amplifification | oyster,clam, codfifish, jellyfifish, shrimp, milk,  and squid, | 10-10^6^  cfu mL^-1^ | 120min | 10 cfu mL^-1^ | (5) |
| A sensitive recombinase aided amplification | fecal and  Fish | — | 240min | 0.1 cfu mL^-1^ | (6) |
| Faraday cage-type aptasensor | oyster  barracuda | 1 -10^8^ cfu mL^-1^ | — | 1.7 cfu mL^-1^ | (7) |
| Label-Free Colorimetric  Method | shrimp | — | — | 6.1× 10^2^ cfu g^-1^ | (8) |
| Multi-colorimetric | mackerel and seaweed | 0-10^4^ cfu mL^-1^ | 35min | 1.12 cfu mL^-1^ | This work |

**REFERENCE**

1. Zhai Y, Meng X, Li L, Liu Y, Xu K, Zhao C, Wang J, Song X, Li J, Jin M. 2021. Rapid detection of Vibrio parahaemolyticus using magnetic nanobead-based immunoseparation and quantum dot-based immunofluorescence. RSC Advances 11:38638-38647.<http://doi.org/10.1039/d1ra07580b>.

2. Hash S, Martinez-Viedma MP, Fung F, Han JE, Yang P, Wong C, Doraisamy L, Menon S, Lightner D. 2019. Nuclear magnetic resonance biosensor for rapid detection of Vibrio parahaemolyticus. Biomedical Journal 42:187-192.<http://doi.org/10.1016/j.bj.2019.01.009>.

3. Kampeera J, Pasakon P, Karuwan C, Arunrut N, Sappat A, Sirithammajak S, Dechokiattawan N, Sumranwanich T, Chaivisuthangkura P, Ounjai P, Chankhamhaengdecha S, Wisitsoraat A, Tuantranont A, Kiatpathomchai W. 2019. Point-of-care rapid detection of Vibrio parahaemolyticus in seafood using loop-mediated isothermal amplification and graphene-based screen-printed electrochemical sensor. Biosensors and Bioelectronics 132:271-278.<http://doi.org/10.1016/j.bios.2019.02.060>.

4. Yonekita T, Morishita N, Arakawa E, Matsumoto T. 2020. Development of a monoclonal antibody for specific detection of Vibrio parahaemolyticus and analysis of its antigen. Journal of Microbiological Methods 173.<http://doi.org/10.1016/j.mimet.2020.105919>.

5. Song S, Wang X, Xu K, Xia G, Yang X. 2019. Visualized Detection of Vibrio parahaemolyticus in Food Samples Using Dual-Functional Aptamers and Cut-Assisted Rolling Circle Amplification. Journal of Agricultural and Food Chemistry 67:1244-1253.<http://doi.org/10.1021/acs.jafc.8b04913>.

6. Feng Z-s, Li J-y, Zhang J-y, Li F-y, Guan H-x, Zhang R-q, Liu H, Guo Q, Shen X-x, Kan B, Ma X-j. 2022. Development and evaluation of a sensitive recombinase aided amplification assay for rapid detection of Vibrio parahaemolyticus. Journal of Microbiological Methods 193.<http://doi.org/10.1016/j.mimet.2021.106404>.

7. Wei W, Lin H, Shao H, Hao T, Wang S, Hu Y, Guo Z, Su X. 2020. Faraday cage-type aptasensor for dual-mode detection of Vibrio parahaemolyticus. Microchimica Acta 187.<http://doi.org/10.1007/s00604-020-04506-1>.

8. Chen X, Wang L, He F, Chen G, Bai L, He K, Zhang F, Xu X. 2021. Label-Free Colorimetric Method for Detection of Vibrio parahaemolyticus by Trimming the G-Quadruplex DNAzyme with CRISPR/Cas12a. Analytical Chemistry 93:14300-14306.<http://doi.org/10.1021/acs.analchem.1c03468>.
